# Pain in children and adolescents with cerebral palsy – a cross-sectional register study of 3545 individuals

**DOI:** 10.1186/s12883-019-1597-7

**Published:** 2020-01-11

**Authors:** Elsa Eriksson, Gunnar Hägglund, Ann I. Alriksson-Schmidt

**Affiliations:** 1 Faculty of Medicine, Lund University, Lund, Sweden; 2 Department of Clinical Sciences Lund, Lund University, Skane University Hospital, Lund, Sweden

**Keywords:** Pain, Cerebral palsy, Children, Youths, Pain intensity

## Abstract

**Background:**

Pain is a common problem for individuals with cerebral palsy (CP). In Sweden, 95% of children and adolescents with CP are followed in a national follow-up programme (CPUP), which includes data on pain. The purpose of this study was to investigate the prevalence of pain based on age, sex, gross motor function and source of report (self or proxy). Pain intensity, pain site, and how much pain disturbed sleep and daily activities were also studied.

**Methods:**

This was a cross-sectional register study based on all participants in CPUP, 4–18-years of age, with data reported in 2017–2018. Gross motor function was classified using the Gross Motor Function Classification System (GMFCS). Logistic regression was used to analyse prevalence of pain and how much pain had disturbed sleep and daily activities in the last four weeks.

**Results:**

In total, 3545 participants (2065 boys) were included. The overall prevalence of pain was 44%. Older age and female sex were associated with higher risk of pain with odds ratios of 1.07 (95% confidence interval (CI) 1.06–1.09) and 1.28 (CI 1.12–1.47), respectively. Pain was most common in the lower extremities. There was no statistically significant difference in prevalence of pain related to source of report. Pain intensity was higher at older ages and higher GMFCS-levels. Hip/thigh pain and abdominal pain were associated with the most intense pain.

Of those who reported pain, pain disturbed sleep for 36% and daily activities for 61%.

**Conclusions:**

Both pain frequency and pain intensity were higher at higher age. Pain intensity increased with increasing GMFCS-level. Two-thirds of all children and adolescents with CP reported that their pain disturbed their daily activities, and one-third reported that pain disturbed their sleep.

## Background

Cerebral palsy (CP) is the terminology for a group of movement disabilities caused by damage or abnormal development of the brain during pregnancy, delivery or the first two years of life. The brain injury is non-progressive and affects movement and posture [1]. CP is the most common cause of impaired motor function among children [2] and reported birth prevalence for CP falls between 1.5–2.7 per 1000 live births [3]. In order to fulfil the criteria of a diagnosis of CP, the disability has to be severe enough to limit activity [1]. The aetiology of CP includes a number of different causes, such as asphyxia, perinatal infections and brain malformations [3].

Numerous comorbidities and secondary conditions are common in CP and may affect performance and/or behaviour. Comorbidities include disturbed sensation, perception, communication, behaviour and epilepsy [1]. Secondary conditions, such as pain and musculoskeletal problems, may arise over time and differ from comorbidities in that they are results of the primary condition and preventable [4].

The severity of symptoms in CP covers a broad range. The most widely used way to classify the level of motor impairment is the Gross Motor Function Classification System (GMFCS) with five ordinal levels. At GMFCS-level I, the child can move freely but is limited in speed, balance and coordination. At level V, the child has severely limited mobility and is unable to stand or sit independently [5].

Pain is one of the most frequent secondary conditions reported in CP, with 30–70% experiencing pain on a regular basis [6–8]. Girls usually report pain more often than boys and the pain intensity appears to be more severe in girls [6, 7, 9] Furthermore, the frequency of pain increases with age [7, 9]. Being in pain can reduce health related quality of life, [10] and participation [8], and it can also cause stress in the parents and families [11]. Many with CP experience pain in the lower extremities but headaches and abdominal pain are also frequently reported [6, 7].

Treatment is generally focused on relieving symptoms, maintaining function, increasing participation in activities and preventing secondary conditions. For example, intrathecal baclofen pumps and botulinum toxin injections may be used to reduce spasticity and physiotherapy to increase mobility. The purpose of both these treatments is to reduce secondary conditions such as contractures [12].

A follow-up programme for people with CP called the Cerebral Palsy Follow-Up Programme (CPUP) was created in Sweden during the 1990s. Approximately 95% of children and adolescents with CP in Sweden are enrolled. In CPUP, children at GMFCS-level I are examined by their physiotherapists annually up to 6 years of age and then every second year. Those at GMFCS-levels II–V are examined twice a year up to 6 years, then once a year. The CPUP assessment contains a questionnaire with items on pain. In addition to a question if the child is in pain, there are items concerning pain sites, pain intensity, source of report (self- or proxy-report) and two items on how much pain has disturbed sleep and daily activities in the past four weeks.

The purpose of this study was to investigate the prevalence of pain in 4–18-year-olds with CP, by age, sex, GMFCS-level and source of report and to analyse pain with regards to pain site, pain intensity and how much pain has disturbed sleep and daily activities in the past four weeks.

## Methods

This was a cross-sectional study based on data from the CPUP register. Information from the latest examinations carried out in 2017–2018 was used. Participants had to be between 4 and 18 years at the time of the examinations in order to be included in the study.

Sex was coded as a dichotomous variable (boy/girl). Age was calculated as whole years using date of birth and date of examination. The GMFCS was used to classify gross motor function, coded as a categorical ordinal variable from I-V. The GMFCS has shown excellent interrater reliability and test-retest reliability [13].

Pain items were reported either by the participant or by a proxy and were recorded as a dichotomous variable (self-report/proxy-report). Reported pain was recorded as a dichotomous variable (yes/no). Furthermore, the participants were asked how much the pain had disturbed their sleep and daily activities during the last four weeks. These were registered as ordinal variables with five levels (not at all, mildly, moderately, quite a bit, considerably). Pain intensity was recorded as an ordinal variable with five levels, which, for the purposes of this study, were recoded into three (mild, moderate, severe) levels. There were 12 different options of pain sites, (head, neck, teeth, back, arms and hands, shoulders, hips and thighs, knees, feet and lower legs, abdomen, skin-pressure wounds, other). Head, neck and teeth were combined into “Head area”, arms and hands were combined into “Upper extremity”. Pain intensity was graded individually for each pain site. For grouped pain sites, highest intensity was used. Missing intensity for a pain site was coded as no pain at that particular site. “Skin and pressure wounds” and “Other” were not included in further analyses due to small numbers.

### Statistical analysis

Descriptive analyses were performed using means and standard deviations (SD) for the continuous variables and raw numbers and percentages for the categorical and ordinal data. Distributions of pain sites and pain intensity are presented using descriptive statistics. In the descriptive analyses, age was divided into groups of two-year spans, thereby compensating for the fact that the participants at GMFCS-level I are examined only every two years after the age of six years. In the remaining statistical analyses, age was not grouped and was included as a continuous variable. For the purposes of this study, the ordinal scales for how much pain disturbed sleep and/or daily activity were recoded as “No” (not at all) and “Yes” (mildly-considerably).

When analysing prevalence of pain, all cases were included. When analysing pain intensity, only the cases with intensity registered were included. The variable “number of pain sites” corresponded to the number of pain sites for each individual where pain was recorded. “Highest pain intensity” was the single highest intensity recorded at any pain site for each individual.

Binary logistic regression was used to regress the independent variables age, sex, GMFCS-level and source of report on presence of pain. Male sex, GMFCS-level I and self-report were set as reference groups. Two separate binary logistic regressions were used to regress age, sex, GMFCS-level, source of report, highest pain intensity and number of pain sites on sleep and daily activities, respectively. Male sex, GMFCS-level I, self-report, mild pain and one pain site were used as reference groups. Logistic regressions were reported as odds ratios (OR) and 95% confidence intervals (CIs). Listwise deletion was used in the binary logistic regression analyses. Analyses were performed using SPSS version 25. Statistical significance was defined as *p* < 0.05.

The study was approved by the Ethics Board at Lund University (LU 433/99).

## Results

In total, 3545 children and adolescents were included (2065 boys, 58.3%), with a mean age of 10.7 years (SD 4.2 years). The distributions of GMFCS-levels, sex and age are presented in Table 1.

**Table 1:** Characteristics of the study sample

Characteristics		Boys n(%)	Girls n(%)	Total n(%)
Age, years	4–5	247 (12.0)	164 (11.1)	411 (11.6)
	6–7	314 (15.2)	230 (15.5)	544 (15.3)
	8–9	297 (14.4)	214 (14.5)	511 (14.4)
	10–11	320 (15.5)	206 (13.9)	526 (14.8)
	12–13	292 (14.1)	189 (12.8)	481 (13.6)
	14–15	266 (12.9)	191 (12.9)	457 (12.9)
	16–17	221 (10.7)	197 (13.3)	418 (11.8)
	18	108 (5.2)	89 (6.0)	197 (5.6)
	Total	2065	1480	3545
GMFCS-level^a^	I	931 (45.1)	650 (43.9)	1581 (44.6)
	II	305 (14.8)	241 (16.3)	546 (15.4)
	III	200 (9.7)	129 (8.7)	329 (9.3)
	IV	291 (14.1)	226 (15.3)	517 (14.6)
	V	338 (16.4)	234 (15.8)	572 (16.1)
	Total	2065	1480	3545
Proxy or self-report	Proxy	1006 (50.4)	689 (47.8)	1695 (49.3)
	Self	992 (49.6)	752 (52.2)	1774 (50.7)
	Total	1998	1441	3439

a *GMFCS* Gross Motor Function Classification System

**Fig. 1:**
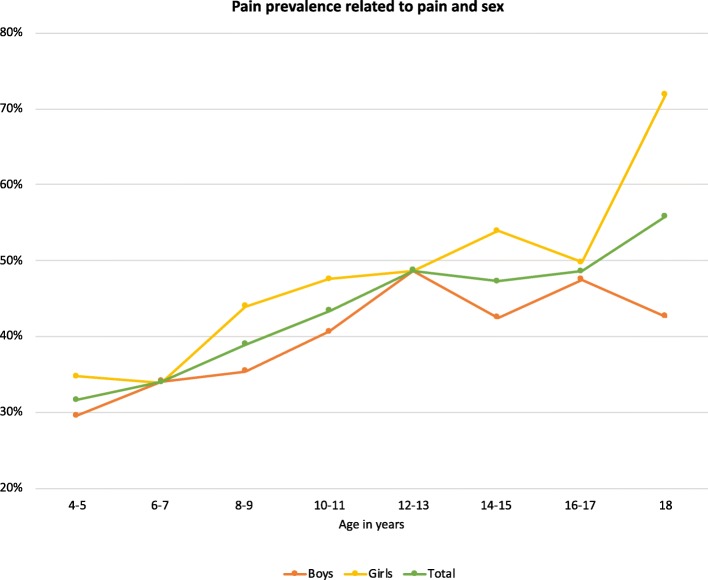
Pain prevalence related to age and sex. *N* = 1505

Pain was reported in 1505 (42.5%) of the participants, 1918 (54.1%) reported no pain and 122 (3.4%) did not answer that specific item. The prevalence of pain was positively associated with age and ranged from 32.8% at 4–5 years to 57.3% at 18 years of age (OR 1.07, 95% CI 1.06–1.09) (Fig. 1). Pain was reported more often in girls than boys (OR 1.28, 95% CI 1.12–1.47) and in children and adolescents at GMFCS-level V (OR 1.74, CI 1.38–2.19). The differences between GMFCS-levels II-IV and level I and source of report (self- versus proxy) were not statistically significant (Table 2).

**Table 2:** Binary logistic regression presenting the odds ratios on pain prevalence for sex, age, Gross Motor Function Classification System level and source of report

	Odds ratio	95% Confidence interval	*P*-value
Girls	1.282	1.116–1.474	< 0.001
Age	1.074	1.055–1.094	< 0.001
GMFCS*			
II	1.139	0.927–1.399	0.216
III	0.976	0.757–1.259	0.852
IV	1.040	0.835–1.294	0.728
V	1.738	1.380–2.190	< 0.001
Self-report	0.967	0.814–1.150	0.705

Reference groups were male sex, GMFCS-level I and proxy-report, respectively. Age was analysed as a continuous variable. *GMFCS = Gross Motor Function Classification System. Included in analysis n = 3411. Missing cases n = 134

**Fig. 2:**
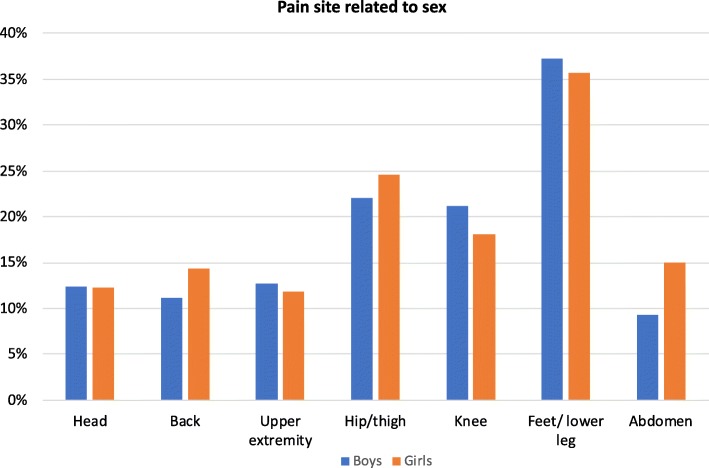
Prevalence of pain for the different pain sites in boys and girls. *N* = 1936

**Fig. 3:**
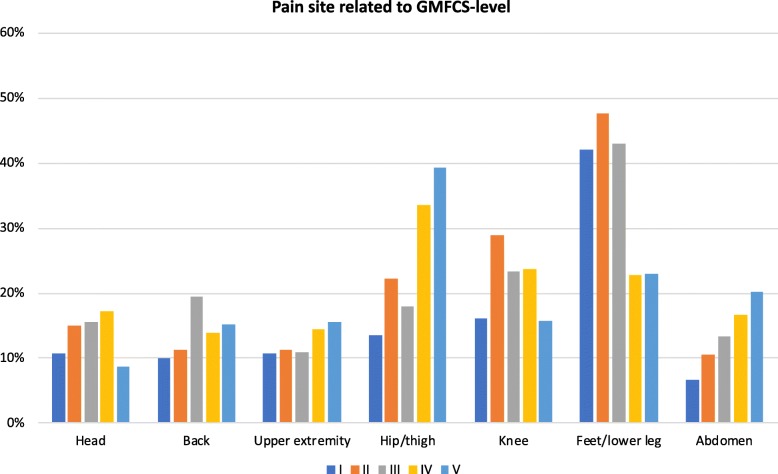
Pain prevalence at different pain sites related to GMFCS-level. Each bar represents the percentage of children with pain related to the total number of children in the same GMFCS-level with pain. GMFCS = Gross Motor Function Classification System

The most common pain site for both boys and girls across all ages was pain in the feet/lower legs followed by hips/thighs and knees (Fig. 2). Abdominal pain and back pain were more common in girls. The most common pain site for children at GMFCS-levels I-III was the feet/lower leg, whereas for those at GMFCS-levels IV-V, the hips/thighs was the most prevalent pain site. Knee pain was most common at GMFCS-levels II-IV. Abdominal pain and pain in the upper extremity were more prevalent at higher GMFCS-level (Fig. 3).

**Fig. 4:**
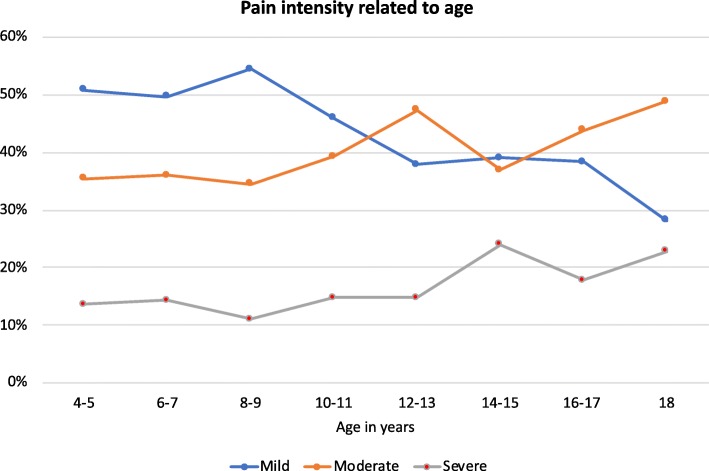
Highest recorded pain intensity related to age. Only those 1118 who have reported pain and pain intensity are included

**Fig. 5:**
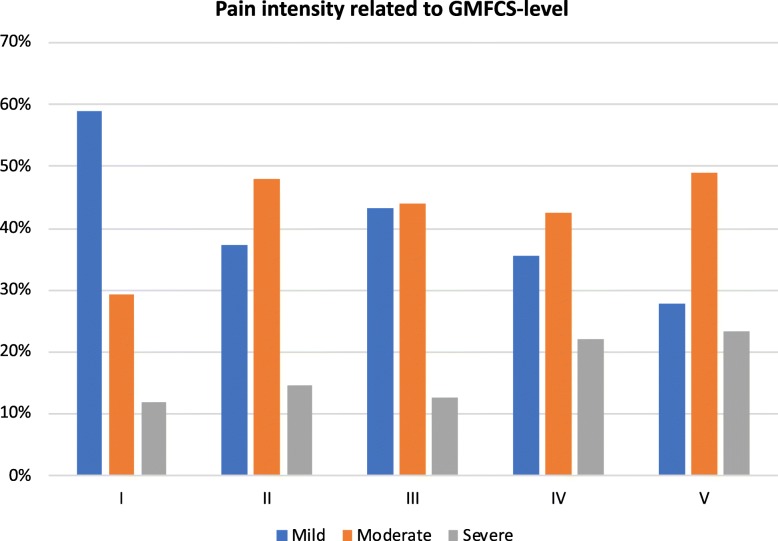
Distribution of highest pain intensity recorded at any pain site related to GMFCS-level. Only those 1118 who reported pain and pain intensity are included. GMFCS = Gross Motor Function Classification System

Of the 1243 participants with reported pain intensity, 796 (64.0%) had pain in one site, 215 (17.3%) in two sites, 133 (10.7%) in three sites, 46 (3.7%) in four sites and 53 (4.2%) in five or more sites. There was more moderate and severe pain, and less mild pain with increasing age (Fig. 4). Mild pain was lower and severe pain higher at higher GMFCS-levels (Fig. 5).

Of those 1050 with data on how their pain disturbed their daily activities, 641 (61.0%) reported that their pain had disturbed their daily activities during the last four weeks (Table 3). A statistically significant higher risk of having pain that disturbed daily activities was seen among those at GMFCS-level V and those with moderate and severe pain intensity (Table 4).

**Table 3:** Pain prevalence, effect on sleep and activities and the highest recorded pain intensity

Item	*Response*	*N (%)*
Do you, or anyone close to you, experience that you are in pain?	Yes	1505 (44.0)
	No	1918 (56.0)
	Total	3423
Has pain affected your daily activities in the last four weeks?	Yes	641 (61.0)
	No	409 (39.0)
	Total	1050
How much has pain affected your daily activities in the last four weeks?	Not at all	409 (39.0)
	Mildly	355 (33.8)
	Moderately	183 (17.4)
	Quite a bit	66 (6.3)
	Considerably	37 (3.5)
	Total	1050
Has pain affected your sleep in the last four weeks?	Yes	371 (35.7)
	No	669 (64.3)
	Total	1040
How much has pain affected your sleep in the last four weeks?	Not at all	669 (64.3)
	Mildly	175 (16.8)
	Moderately	119 (11.4)
	Quite a bit	53 (5.1)
	Considerably	24 (2.3)
	Total	1040
Highest level of pain intensity recorded at any pain site	None	1918 (63.2)
	Mild	486 (16.0)
	Moderate	449 (14.8)
	Severe	183 (6.0)
	Total	3036

**Table 4:** Results from binary logistic regression of pain effect on sleep and daily activities

	Sleep			Daily activities		
	Odds ratio	95% CI	P-value	Odds ratio	95% CI	P-value
Sex	1.062	0.788–1.432	0.692	1.027	0.782–1.349	0.847
Age	0.999	0.960–1.039	0.949	1.001	0.965–1.039	0.937
GMFCS* II	1.212	0.786–1.869	0.384	1.230	0.839–1.803	0.289
GMFCS III	0.869	0.490–1.542	0.632	1.156	0.718–1.862	0.550
GMFCS IV	2.084	1.304–3.329	0.002	1.219	0.786–1.888	0.376
GMFCS V	3.499	2.156–5.679	< 0.001	1.936	1.213–3.090	0.006
Source of report	0.770	0.529–1.120	0.171	0.932	0.664–1.308	0.683
Number of pain sites						
2	1.206	0.820–1.773	0.342	1.128	0.789–1.613	0.510
3	1.534	0.976–2.412	0.064	1.534	0.984–2.390	0.059
4	1.680	0.807–3.496	0.165	1.287	0.601–2.759	0.516
≥ 5	2.801	1.350–5.809	0.006	2.299	0.965–5.479	0.060
Pain intensity						
Moderate	3.106	2.194–4.397	< 0.001	2.268	1.688–3.047	< 0.001
Severe	8.641	5.509–13.554	< 0.001	5.843	3.538–9.648	< 0.001

Reference groups: male sex, GMFCS-level I, proxy-report, one pain site and mild intensity respectively. Age was analysed as a continuous variable. * *GMFCS*= Gross Motor Function Classification System. Included in sleep analysis *n* = 996. Included in activity analysis *n* = 1007

Of those 1040 who answered how their pain affected their sleep, 371 (35.7%) reported that their pain had disturbed their sleep during the last four weeks. Of those whose sleep was disturbed, a majority reported moderate or more severe effects (Table 3). Sleep was more often disturbed in those at GMFCS-levels IV-V, in those with pain at five or more sites and in those with moderate or severe pain (Table 4).

## Discussion

This study investigated the prevalence of pain and pain intensity in 4 to 18-year-olds with CP in Sweden. A large proportion of the studied population experienced pain, with girls and older age being risk factors. There was also a statistically significant increased risk for children at GMFCS-level V when compared to GMFCS-level I. Pain was more frequent in the lower extremities. More severe pain was noted at higher GMFCS-levels and in those who reported hip/thigh pain and abdominal pain. Two-thirds reported that pain disturbed their daily activities and one-third that pain had disturbed their sleep in the past four weeks.

Pain is difficult to study due to its subjective and changing nature. It is therefore not surprising that studies report different findings and possible explanations for different prevalence of pain across studies using different study designs and inclusion criteria. The overall prevalence of pain in this study was 44%, which is higher than in previous research conducted on the CPUP register, which showed a pain prevalence of 32% [7]. This could be explained by the three year higher mean age in the present study. Nevertheless, a 44% prevalence of pain in children and adolescents with CP is still lower than in many international studies, where pain prevalence up to 60–70% has been reported [6, 14]. Part of the explanation for the lower pain prevalence in these Swedish studies could be that CPUP is population-based and does not only include those who seek medical care. The lower frequency of pain in Sweden could also, to some extent, be a consequence of the CPUP-programme resulting in fewer children with painful dislocated hips, severe contractures and scoliosis [15].

Consistent with other studies, girls and older age are risk factors for pain [6, 7, 9] The pain prevalence for girls aged 18 years was much higher than for 16–17-year-olds. The distribution of GMFCS-levels was similar across all age-groups, including 18-year-olds, and could not explain the sudden divergence.

In accordance with other studies, pain in the lower extremities was the most common pain site regardless of sex, age and GMFCS-level [6, 8]. The distribution of pain sites differed by GMFCS-levels. This is probably related to the differences in ambulatory status, with different body parts experiencing stress, depending on the gross motor function. At GMFCS-levels I-II, where children are ambulatory, more stress and body weight are placed on the feet and lower legs. Walking ability is more affected at GMFCS-level III, sometimes resulting in a crouch gait and more stress on the knees. At GMFCS-levels IV-V, mobility is more severely affected, with stress on hips and thighs from prolonged sitting in the same position.

Hip/thigh pain and abdominal pain were the body sites with most severe pain reported. The GMFCS-levels most often reported for these sites were GMFCS-levels IV-V. Abdominal pain can be caused by constipation and gastroesophageal reflux, which is more frequent at higher GMFCS-levels [16, 17]. Even though pain prevalence did not change with GMFCS-levels, those at higher GMFCS-levels reported more intense pain, making this a group of special concern. It should be noted, however, that proxy reports are more likely at higher GMFCS-levels. Thus, the pain intensity reported at higher GMFCS-levels in particular was that perceived by parents. It is possible that proxies over or under report the intensity of pain and results should be interpreted with that in mind. Pain intensity was also higher at older ages. This demonstrates the importance of treating and preventing pain, as not only pain frequency but also pain severity is a more common problem at older ages.

Six of ten children with pain reported that the pain disturbed their daily activities. Being in pain can restrict what activities are performed. Riquelme et al. [18] found that children with chronic pain and CP to a lesser extent than their typically developing peers participated in physical activities and that pain restricted the level of activity. Reducing pain might therefore allow a greater freedom and more possibilities to engage in activities for the person. Few studies have investigated the relationship between pain and activity. Penner et al. showed a 47–55% overall prevalence of pain, with approximately 25% reporting that pain affected activity [8]. In the Penner et al.’s study, the lowest limit for affected activity was phrased as “moderate pain that prevents a few activities”, whereas the present study allowed grading of pain and effects on activity separately, which might explain the differences in results.

About one-third reported that pain disturbed their sleep. Of those, more than half experienced moderate or more severe effects on sleep. Sleep deprivation is linked to a number of negative health effects, such as lowered cognitive function and behavioural problems [19]. There is also some evidence that sleep deprivation could contribute to more pain by inducing a state of hyperalgesia [20].

The effects pain had on daily activities and sleep were not related to sex, age or whether the information was self- or proxy reported. It is plausible that the characteristics of pain, such as type, duration, site and intensity is what predicts how and to what extent pain will influence sleep or activities. One explanation as to why individuals at higher GMFCS-levels had higher risks of pain disturbing daily activities and sleep could be that many of them are unable to change position themselves, meaning that they have limited ability to avoid or get out of painful positions while lying and sitting.

Using a cross-sectional register study limits the information available for analysis. It was not possible to analyse pain related to duration (acute versus chronic) or the reason for using proxy-reporting. In this study, we did not observe any statistically significant differences in pain prevalence based on source of report, but there could be differences regarding intensity and pain site. Using a cross sectional study, it is not possible to draw any conclusions regarding cause and effect.

The participants self-reported their level of pain and how much it disturbed daily activities and sleep, on a subjective scale. However, most pain scales are subjective, such as the Visual Analog Scale (VAS) [21]. The subjective grading allows us to study the participant’s experience, rather than objective type of pain. When the items were proxy-reported, this effect is of course reduced.

Data on pain medication were not available which would have been informative and is an important topic for future studies. Children and adolescents who had received intrathecal baclofen and botulinum toxins were included in the sample, as we wanted to investigate the prevalence of pain. Although these treatments are primarily prescribed to reduce spasticity, it is possible that they also reduce pain.

The major strength of this study is that the large sample was drawn from the total population of children with CP, resulting in a reduced risk of selection bias. The level of participation was high for all ages included and among the variables used there were little missing data. This was the first study using new data from CPUP about how pain affects sleep and daily activities, which is useful for understanding how the pain is perceived by the participants.

## Conclusion

Pain is common in children and adolescents with CP. Older age and being a girl are risk factors for pain. Pain intensity was higher at higher age and in children at higher GMFCS-levels. Pain in the lower extremities was most common. Pain often disturbs daily activities and sleep.

## Data Availability

Data used in this study are stored at the National Quality Register CPUP http://rcsyd.se/anslutna-register/cpup. Data are not publicly available and permission to extract data can be obtained from the register holder.

## Abbreviations

CI: Confidence interval
CP: Cerebral palsy
CPUP: Cerebral Palsy Follow-Up Programme
GMFCS: Gross Motor Function Classification System
OR: Odds ratio
SCPE: Surveillance of Cerebral Palsy in Europe
SD: Standard deviation
